# Bisphenol A Exposure Disrupts Organelle Distribution and Functions During Mouse Oocyte Maturation

**DOI:** 10.3389/fcell.2021.661155

**Published:** 2021-03-23

**Authors:** Meng-Hao Pan, Yu-Ke Wu, Bi-Yun Liao, Hui Zhang, Chan Li, Jun-Li Wang, Lin-Lin Hu, Baohua Ma

**Affiliations:** ^1^Key Laboratory of Animal Biotechnology, Ministry of Agriculture, College of Veterinary Medicine, Northwest A&F University, Yangling, China; ^2^Life Sciences Institute, Zhejiang University, Hangzhou, China; ^3^The Affiliated Hospital of Youjiang Medical University for Nationalities, Baise, China

**Keywords:** BPA, oocyte, meiosis, organelles, mitochondria, ER stress, lysosome

## Abstract

Bisphenol A (BPA) is one of the ubiquitous environmental endocrine disruptors (EEDs). Previous studies have shown that the reproduction toxicity of BPA could cause severe effects on the mammal oocytes and disturb the quality of mature oocytes. However, the toxic effects of BPA on the organelles of mouse oocytes have not been reported. In this study, to investigate whether BPA can be toxic to the organelles, we used different concentrations of BPA (50, 100, and 200 μM) to culture mouse oocytes *in vitro*. The results showed that 100 μM BPA exposure could significantly decrease the developmental capacity of oocytes. Then, we used the immunofluorescence staining, confocal microscopy, and western blotting to investigate the toxic effects of BPA on the organelles. The results revealed that mitochondrial dysfunction is manifested by abnormal distribution and decreased mitochondrial membrane potential. Moreover, the endoplasmic reticulum (ER) is abnormally distributed which is accompanied by ER stress showing increased expression of GRP78. For the Golgi apparatus, BPA-exposed dose not disorder the Golgi apparatus distribution but caused abnormal structure of Golgi apparatus, which is manifested by the decrease of GM130 protein expression. Moreover, we also found that BPA-exposed led to the damage of lysosome, which were shown by the increase of LAMP2 protein expression. Collectively, our findings demonstrated that the exposure of BPA could damage the normal function of the organelles, which may explain the reduced maturation quality of oocytes.

## Introduction

In recent years, with the continuous development of human society, the contradiction between rapidly rising pollution levels and human health has become increasingly prominent. Excessive exposure to environmental pollutants can induce severe health problems, including reproductive health problems, which have attracted increased attention in recent years. This situation is being followed closely by scholars at home and worldwide (Vandenberg et al., 2007; Kabir et al., 2015). Environmental endocrine disruptors (EEDs) are a category of common environmental pollutants. The United States Environmental Protection Agency defines EED as “a substance that disrupts the synthesis, secretion, transport, binding, or attenuation of natural hormones needed to maintain homeostasis, reproduction, development, and normal behavior” (Kavlock et al., 1996). EEDs have attracted the attention of various research institutions because they may contribute to the development of endocrine system disorders in mammals and interfere with normal reproductive functions (Lucaccioni et al., 2020; Shi et al., 2021).

Bisphenol A (BPA) is a common EED that is often used as a synthon of polycarbonate plastics and epoxy resins (Rubin, 2011). It can be detected in chemical products, such as adhesives and surface coatings, and has an estrogen-like effect (Alonso-Magdalena et al., 2012). Excessive exposure to BPA may affect the health of the reproductive system, immune system, and neuroendocrine system of mammals (Chen et al., 2017; Ma et al., 2019). Zhang et al. (2017) carried out an *in vivo* exposure experiment in mice and found that certain concentrations of BPA treatment can disrupt spindle arrangement, chromosome synapsis, and kinetochore microtubule assembly, which impacts the excretion of the first polar body, destroys the meiosis process, and ultimately damages the reproductive capacity of mammals. Furthermore, an *in vitro* exposure experiment by Li and Zhao (2019) revealed that BPA has certain reproductive toxicity and adversely affects the oocyte maturation. However, the mechanism of how BPA retards oocyte development remains unclear, and there are limited studies investigating the toxic effects of BPA on the organelle function of mammalian oocytes.

Assuring the maturation quality of oocytes is the basis of reproduction in all mammals, the maturation quality of the oocyte has a significant influence on subsequent early embryonic development (Sen and Caiazza, 2013; Keefe et al., 2015). Maintaining the normal distribution and function of organelles in oocytes is essential for oocyte maturation, which include the proper distribution of mitochondria, endoplasmic reticulum, Golgi apparatus, lysosomes, and other organelles in the cytoplasm and the maintenance of normal physiological activity (Sirard et al., 2006; Mao et al., 2014). Oocyte mitochondria are the major source of ATP during preimplantation embryonic development (Babayev and Seli, 2015). A study by Duan et al. (2015) showed that excessive exposure to harmful substances not only leads to the obstruction of oocyte development but also leads to abnormal mitochondrial distribution in the oocyte, which ultimately affects the maturation quality of the oocyte. To maintain oocyte and embryo development, endoplasmic reticulum is the important place to fold the functional proteins properly (Lin et al., 2019). Lysosomes are membranous organelles that are produced by Golgi apparatus and closely related to the functions of the endoplasmic reticulum and mitochondria and are involved in autophagy (Bajaj et al., 2019; Miao et al., 2019).

There are many studies confirming that BPA has certain reproductive toxicity; however, few studies have reported the effect of BPA on oocyte organelles. In this study, we intended to explore the potential toxic effects of BPA on organelles. The results showed that a 100 μM BPA treatment had some effect on the extrusion rate of the first polar body and the distribution and function of the organelles, which may explain the reduced maturation quality of oocytes following BPA exposure.

## Materials and Methods

### Chemicals and Antibodies

Pregnant horse serum gonadotropin (PMSG) was purchased from Ningbo No.2 hormone factory (Zhejiang, China). Mito-Tracker Red, Mitochondrial membrane potential assay kit with JC-1, ER-Tracker Red, Golgi-Tracker Red, and Lyso-Tracker Red were purchased from Beyotime Biotechnology (Shanghai). Rabbit polyclonal anti-LAMP2 antibody and rabbit polyclonal anti-GRP78 were purchased from Cell Signaling Technology (Danvers, MA, United States). Rabbit monoclonal anti-LAMP2 antibody and rabbit monoclonal anti-GRP78 antibody were purchased from Abcam (Cambridge, United Kingdom). Alexa Fluor 594 goat anti-rabbit antibody was purchased from Zhongshan Golden Bridge Biotechnology (Beijing). All other chemicals and reagents were purchased from Sigma-Aldrich Corp., unless otherwise stated.

### Ethics Statement

We followed the guidelines of the Institutional Animal Care and Use Committee of the College of Veterinary Medicine, Northwest A&F University (No. 2018011212) to conduct the operations. These mice were housed in considerably ideal conditions which consisted of controlled temperature, regular diet, and appropriate light.

### BPA Treatment

Bisphenol A was dissolved in DMSO to prepare a 150 mM stock solution. Taking the research concentration of BPA in porcine oocytes as a reference, we choose the corresponding low concentration for the experiment. We diluted the stock solution in M2 medium to a final working concentration of 50, 100, and 200 μM. For the oocytes collection, mice were intraperitoneally stimulated with 5 IU of PMSG and were then sacrificed by cervical dislocation after 48 h. The oocytes were placed at 37°C with an atmosphere of 5% CO_2_ and cultured in different concentrations of BPA and to different time points for future experiments.

### Immunofluorescent Staining

Oocytes were fixed in 4% paraformaldehyde for 30 min at room temperature, then permeabilized with 0.5% Triton X-100 for 20 min at room temperature, and blocked in blocking buffer (1% BSA-supplemented PBS) at room temperature for 1 h. For GRP78 and LAMP2 staining, the oocytes after blocking were incubated with rabbit polyclonal anti-LAMP2 antibody and rabbit polyclonal anti-GRP78 (1:100) at 4°C overnight, and then the oocytes were washed by wash buffer (0.1% Tween 20 and 0.01% Triton X-100) three times (2 min each time). Next the oocytes were labeled with Alexa Fluor 594 goat anti-rabbit antibody (1:100) at room temperature for 1 h and then washed by wash buffer for three times (2 min each time). Finally, oocytes were incubated with Hoechst 33342 at room temperature for 10 min. After staining, samples were mounted on glass slides and observed with a confocal laser-scanning microscope (Zeiss LSM 800 META, Germany).

### Mito-Tracker Red, ER-Tracker Red, and Lyso-Tracker Red Staining

Oocytes were incubated with ER-Tracker Red (1:3000), Mito-tracker Red (1:2000), or Lyso-tracker Red (1:20000) in M2 medium for 30 min at 37°C and 5% CO_2_. Then, the oocytes were washed 3 times with M2 medium, and finally, the samples were examined with a confocal laser-scanning microscope (Zeiss LSM 800 META, Germany) and confocal scanning laser microscope (Nikon Eclipse Ti, Tokyo, Japan).

### Golgi-Tracker Red Staining

Oocytes were incubated with 1% pronase for 4 min in order to remove the zona pellucida, and then, they were incubated with Golgi-tracker Red (1:100) in M2 medium for 30 min at 37°C and 5% CO_2_. Then, the oocytes were washed 3 times with M2 medium, and finally, the samples were examined with a confocal scanning laser microscope (Nikon Eclipse Ti, Tokyo, Japan).

### Western Blot Analysis

Approximately 200 mouse oocytes were placed in Laemmli sample buffer and heated at 95°C for 10 min. Proteins were separated by SDS-PAGE at 80 V for 45 min and 120 V for 2 h and then electrophoretically transferred to polyvinylidene fluoride (PVDF) membranes (Millipore, Billerica, MA, United States) at 20 V for 3 h. After transfer, the membranes were then blocked with TBST (TBS containing 0.1% Tween 20) containing 5% non-fat milk powder at room temperature for 1 h. After blocking, the membranes were incubated with rabbit monoclonal anti-LAMP2 antibody (1:500) and rabbit monoclonal anti-GRP78 antibody (1:1000) or rabbit monoclonal anti-GAPDH antibody (1:2000) at 4°C overnight. After washing five times in TBST (5 min each), membranes were incubated at 4°C overnight with HRP-conjugated Pierce Goat anti-Rabbit IgG (1:5000). After washing for 5 times, the membranes were visualized using chemiluminescence reagent (Millipore, Billerica, MA).

### Statistical Analysis

At least three biological replicates were performed for each analysis. The fluorescence intensity and the Western blotting band gray intensity were measured by Image J. All analyses were performed using GraphPad Prism7.00 software (GraphPad, CA, United States). Results of *P* < 0.05 were considered statistically significant (differences *P* < 0.05 denoted by ^∗^, *P* < 0.01 denoted by ^∗∗^, *P* < 0.001 denoted by ^∗∗∗^, and *P* < 0.0001 denoted by ^****^). The results were endowed as means ± SEM.

## Results

### Effects of BPA on the Developmental Competence of Mouse Oocytes

**FIGURE 1:**
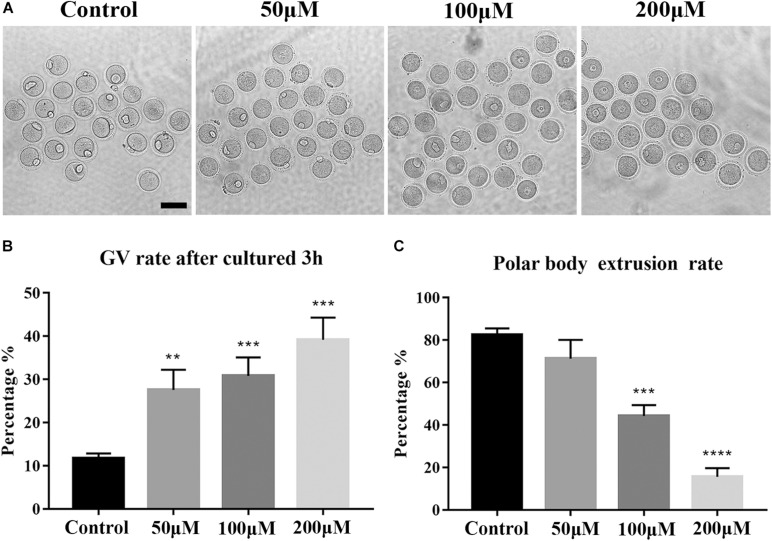
Effects of BPA on the developmental competence of mouse oocytes. **(A)** DIC images of the oocyte polar body extrusion in control, 50, 100, and 200 μM BPA treatment groups. Bar = 100 μm. **(B)** Rate of GV period after 3 h culture of the control group and BPA treatment group. **, significant difference (*P* < 0.01); ***, significant difference (*P* < 0.001). **(C)** Rate of polar body extrusion after 12 h culture of the control group and BPA treatment group. ***, significant difference (*P* < 0.001); ****, significant difference (*P* < 0.0001).

To investigate the toxic effects of BPA on mouse oocytes, we first examined the maturation of oocytes with a rising concentration gradient which is exposed with 50, 100, and 200 μM BPA, respectively. We observed polar body extrusion, and the results showed that most oocytes could extrude first polar body in the control group and 50 μM BPA treatment group; however, in the 100 and 200 μM treatment groups, most oocytes had developmental block (Figure 1A). The quantitative results also confirmed this phenotype (Rate of GV rate: Control group: 11.71 ± 1.157%, *n* = 233, 50 μM: 27.57 ± 4.665%, *n* = 194, *P* < 0.01, 100 μM: 30.81 ± 4.307%, *n* = 202, *P* < 0.001, 200 μM: 39.2 ± 5.124%, *n* = 268, *P* < 0.001, Figure 1B. Rate of first polar body extrusion: Control group: 82.56 ± 2.1984%, *n* = 134, 50 μM: 71.29 ± 8.807%, *n* = 165, *P* > 0.05, 100 μM: 44.29 ± 5.053%, *n* = 145, *P* < 0.001, 200 μM: 15.62 ± 4.07%, *n* = 94, *P* < 0.0001, Figure 1C). These results suggested that BPA exposure could reduce the developmental competence of mouse oocytes in the dose-dependent manner. Furthermore, we next selected 100 μM as the working concentration in the subsequent study.

### Effects of BPA on Mitochondrial Distribution and Functions During Mouse Oocyte Maturation

**FIGURE 2:**
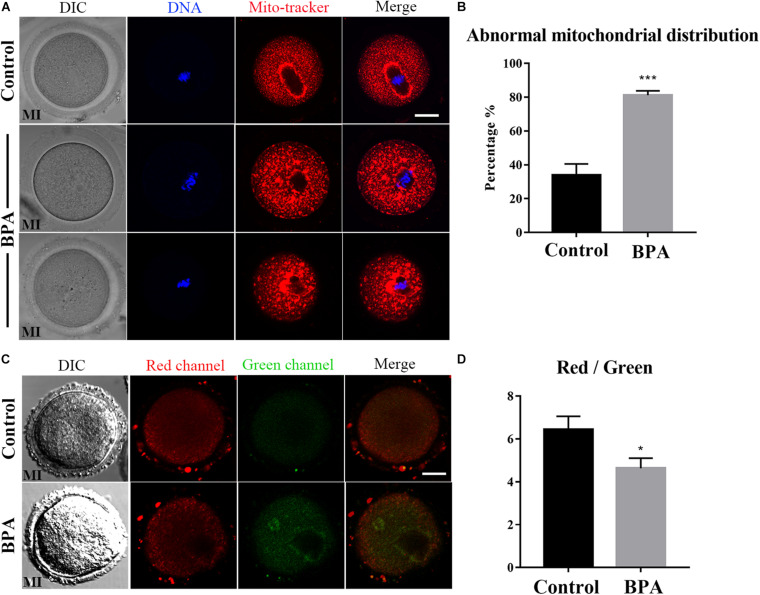
Effects of BPA on mitochondrial distribution and functions during mouse oocyte maturation. **(A)** Representative images of mitochondrial distribution in the oocyte cytoplasm in the control group and BPA treatment group. Red, Mito; Blue, DNA. Bar = 20 μm. **(B)** Abnormal distribution of mitochondrial significantly increased in the BPA treatment oocytes compared with the control oocytes. ***, significant difference (*P* < 0.001). **(C)** The typical picture for JC1 red channel and green channel after BPA treatment. Bar = 20 μm. **(D)** The JC1 signal (red/green ratio) after BPA treatment compare with the control group, the JC-1 red/green fluorescence ratio was significantly reduced in BPA treatment group. *, significant difference (*P* < 0.05).

To further explore the effects of BPA on the maturation of mouse oocytes, we then explored the effects of BPA on the organelles. We first examined the distribution of mitochondria in BPA treatment oocytes. As Figure 2A, in the control group, the mitochondria are evenly distributed in the cytoplasm and accumulated at the chromosome periphery in oocytes; however, in BPA treatment oocytes, mitochondria appeared as clumped aggregation distribution in cytoplasm. We compared the rate of abnormal mitochondrial distribution between the control group and BPA treatment group; the BPA treatment group was much higher than the control group (33.93 ± 6.61%, *n* = 55 vs. 81.17 ± 2.596%, *n* = 43, *P* < 0.001, Figure 2B). We also examined the mitochondrial membrane potential, and the results showed that BPA treatment could cause the alterations of mitochondrial membrane potential (MMP) by JC-1 staining. The fluorescence intensity of JC-1 red channel was decreased, and green channel was increased compared with the control group (Figure 2C). We also calculated the ratio for red/green fluorescence intensity, and the results also confirmed this (control group: 6.444 ± 0.615, *n* = 9; BPA treatment group: 4.627 ± 0.477, *n* = 18, *P* < 0.05, Figure 2D). These results indicated that BPA exposure adversely affected mitochondria distribution and functions during mouse oocytes maturation.

### Effects of BPA on Endoplasmic Reticulum Distribution and Functions During Mouse Oocyte Maturation

**FIGURE 3:**
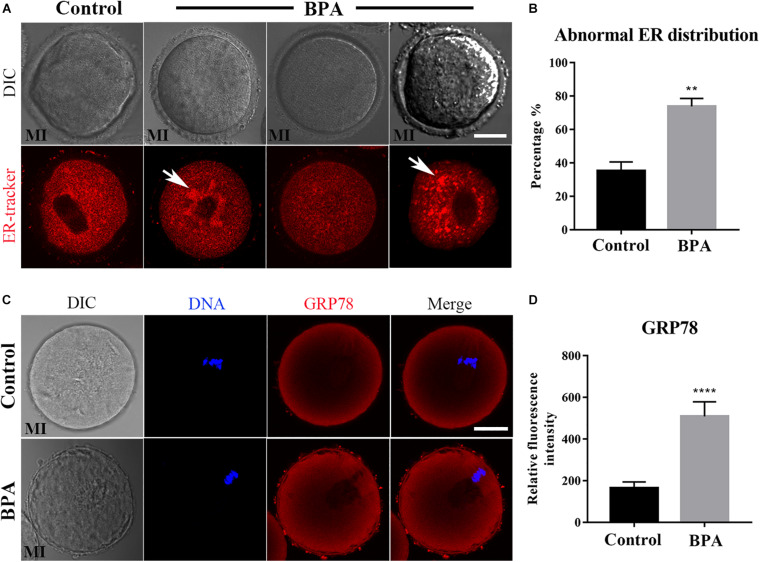
Effects of BPA on endoplasmic reticulum distribution and functions during mouse oocyte maturation. **(A)** Representative images of ER distribution in the oocyte cytoplasm in the control group and BPA treatment group. In BPA treatment oocytes, ER agglomerated in the cytoplasm (white arrow). Red, ER. Bar = 20 μm. **(B)** Abnormal distribution of ER significantly increased in the BPA treatment oocytes compared with the control oocytes. **, significant difference (*P* < 0.01). **(C)** Representative images of GRP78 intensity in the oocyte cytoplasm in the control group and BPA treatment group. Red, GRP78 antibody; Blue, DNA. Bar = 20 μm. **(D)** The fluorescence intensity of GRP78 in the BPA treatment group was significantly increased compared with the control group. ****, significant difference (*P* < 0.0001).

As the ER–mitochondrial interaction is important for the cellular vital movement, we then examined the ER distribution in the BPA-exposed oocytes. As shown in Figure 3A, in the control oocytes, the ER was evenly distributed in the cytoplasm and accumulated at the chromosomes periphery; however, three abnormal distributions appeared in the BPA-exposed oocytes: ER abnormal agglomerated chromosome periphery, the accumulation of ER around the chromosomes disappeared, or ER agglomerated in cytoplasm. The statistical analysis showed that the abnormal distribution of ER increased significantly in the BPA treatment group (35.37 ± 5.228%, *n* = 55 vs. 73.85 ± 4.774%, *n* = 47, *P* < 0.01, Figure 3B). The ER localization pattern indicated that its functions might be disturbed in the BPA-exposed oocytes. To further confirm the effects of BPA on ER, we stained the ER stress marker protein GRP78 to examine the ER stress level. As shown in Figure 3C, we found a significant increase of GRP78 signals in the BPA treatment group, and the statistical analysis for the fluorescent signals also confirmed our findings (164.8 ± 29.74, *n* = 17 vs. 509.3 ± 70.07, *n* = 14, P < 0.0001, Figure 3D). These results indicate that BPA-exposed caused abnormal ER distribution and further led to ER-stress in mouse oocytes.

### Effects of BPA on Golgi Apparatus Distribution During Mouse Oocyte Maturation

**FIGURE 4:**
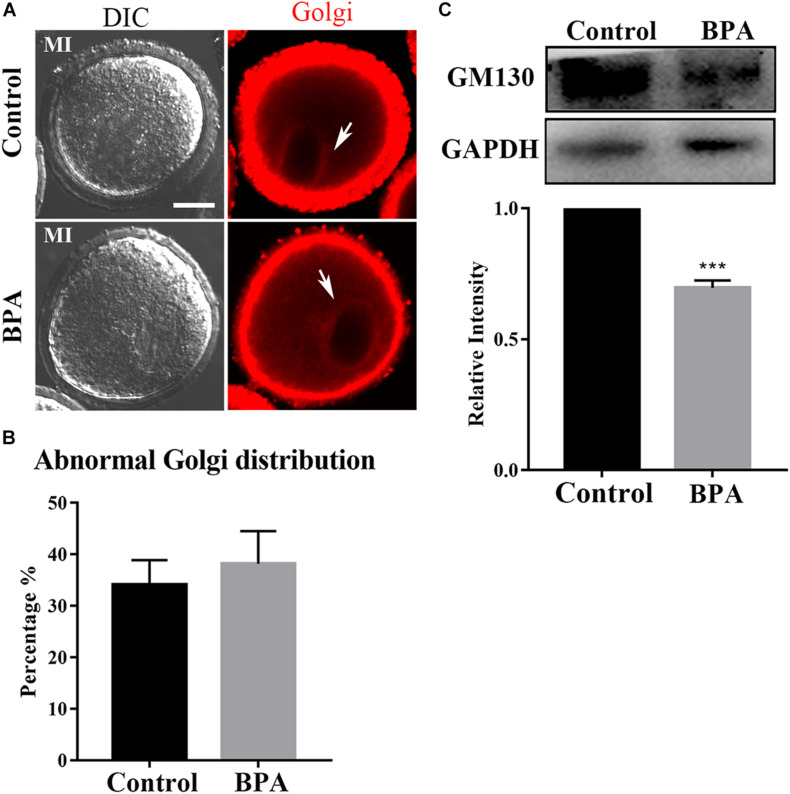
Effects of BPA on Golgi apparatus distribution during mouse oocyte maturation. **(A)** Representative images of Golgi apparatus distribution in the control group and BPA treatment group. Red, Golgi. Bar = 20 μm. **(B)** Abnormal distribution of Golgi apparatus no significant difference in the BPA treatment oocytes compared with the control oocytes. *P* > 0.05. **(C)** Western blot analysis for GM130 expression in the control group and BPA treatment group. Relative intensity of GM130 and GAPDH was assessed by densitometry. ***, significant difference (*P* < 0.001).

Since the protein needs to be processed, sorted, and delivered by Golgi apparatus, we next examined the Golgi apparatus distribution using Golgi-Tracker in the BPA-exposed oocytes. As shown in Figure 4A, there is no difference in the distribution of Golgi apparatus in the control group and BPA treatment group, and the statistical analysis for the fluorescent signals also confirmed our findings (34.08 ± 4.823%, *n* = 37 vs. 38.18 ± 6.355%, *n* = 46, *P* > 0.05, Figure 4B). Since BPA exposure has no effect on the distribution of Golgi apparatus, we further examined whether BPA has an effect on the structure of Golgi apparatus. As GM130 protein participates in the maintenance of Golgi apparatus, we examined the protein expression of GM130 and found that BPA exposure significantly decreased the GM130 protein level compared with the control group (1 vs. 0.696 ± 0.029, *P* < 0.001, Figure 4C). These results indicate that BPA exposure caused abnormal structure of Golgi apparatus but does not affect the distribution of Golgi apparatus in mouse oocytes.

### Effects of BPA on Lysosome Functions During Mouse Oocyte Maturation

**FIGURE 5:**
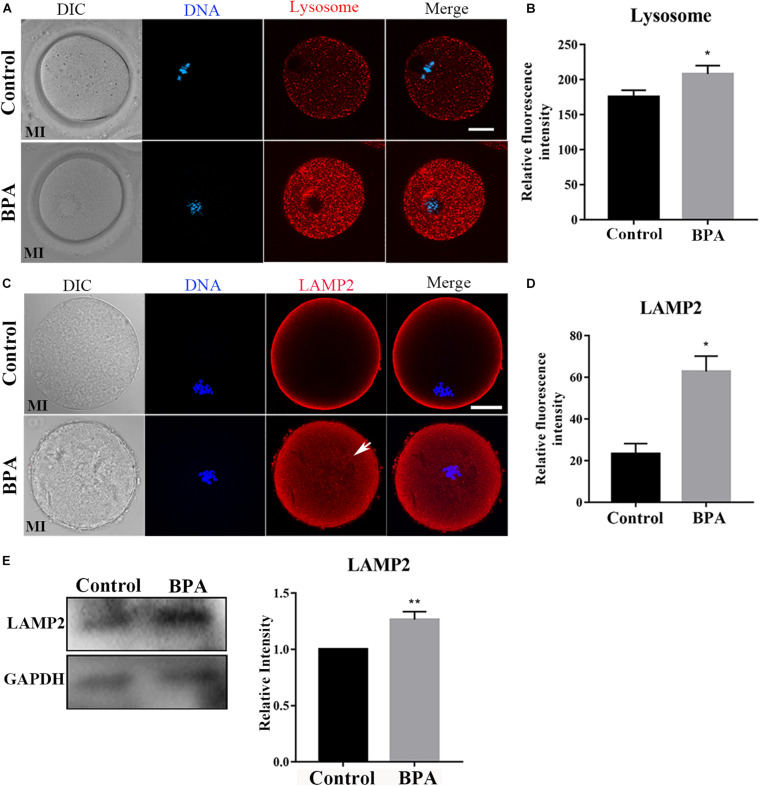
Effects of BPA on lysosome functions during mouse oocyte maturation. **(A)** Representative images of lysosome distribution in the oocyte cytoplasm in the control group and BPA treatment group. Red, lysosome; Blue, DNA. Bar = 20 μm. **(B)** The fluorescence intensity of lysosome significantly increased in the BPA treatment oocytes compared with the control oocytes. *, significant difference (*P* < 0.05). **(C)** Representative images of LAMP2 intensity in the oocyte cytoplasm in the control group and BPA treatment group. Red, LAMP antibody; Blue, DNA. Bar = 20 μm. **(D)** The fluorescence intensity of LAMP2 in the BPA treatment group was significantly increased compared with the control group. *, significant difference (*P* < 0.05). **(E)** Western blot analysis for LAMP2 expression in the control group and BPA treatment group. Relative intensity of LAMP2 and GAPDH was assessed by densitometry. **, significant difference (*P* < 0.01).

As lysosomes are formed on the trans-face of Golgi apparatus, to further confirm the effects of BPA on organelles during mouse oocyte maturation, we detected the lysosome by using Lyso-Tracker and lysosomal marker protein LAMP2 in the BPA-exposed oocytes. As shown in Figure 5A, in the BPA treatment group, the punctate signals of Lyso-Tracker were stronger compared with the control group, and the statistical analysis for the fluorescent signals also confirmed our findings (175.4 ± 9.432, *n* = 21 vs. 208 ± 12.09, *n* = 22, *P* < 0.05, Figure 5B). The results of LAMP2 analysis were similar to Lyso-Tracker; as shown in Figure 5C, we found that the signal of LAMP2 in BPA exposure oocytes was stronger compared with the control oocytes, and the statistical analysis for the fluorescent signals also confirmed this (23.27 ± 4.962, *n* = 4 vs. 62.81 ± 7.424, *n* = 4, *P* < 0.05, Figure 5D). We also examined the protein expression of LAMP2 and found that BPA exposure significantly increased LAMP2 protein level compared with the control group (1 vs. 1.265 ± 0.070, *P* < 0.01, Figure 5E). These results indicate that BPA exposure caused adverse effects on the lysosome functions in mouse oocytes.

## Discussion

The toxic effects of BPA on oocyte maturation have been reported previously. However, the specific underlying mechanism remains unknown. To better understand the effect of BPA treatment on oocyte development at the cytobiological level, we used mouse oocyte samples to detect the excretion rate of the polar body, mitochondria, endoplasmic reticulum, Golgi apparatus, and lysosomes. The results of the statistical analysis showed that a 100 μM BPA treatment had some effect on the excretion rate of the first polar body and the distribution and function of the organelles, which may explain the reduced maturation quality of oocytes following BPA-exposed.

During the development of oocytes, the excretion of the first polar body signals the completion of the first meiosis and indicates that the oocyte has reached the state of nuclear maturation (Sen and Caiazza, 2013). Therefore, the excretion rate of the first polar body is a good indicator of the developmental ability of a mouse oocyte (Sato and Tokmakov, 2020). First, we detected the effect of BPA treatment on the extrusion of the first polar body in a mouse oocyte. The results showed that the extrusion rate of the first polar body was significantly reduced in the BPA-treated group. This indicated that BPA treatment disrupted the first meiosis in the mouse oocyte, which resulted in a poorer quality oocyte. This finding is consistent with the results of an *in vitro* BPA exposure experiment in pig oocytes by Wang et al. (2016).

Oocytes need adenosine triphosphate (ATP) to carry out meioses and other vital activities, and oocyte mitochondria are the major source of ATP during preimplantation embryonic development (Babayev and Seli, 2015). During the first meiosis of the oocyte, the spindle that is formed by microtubules binds to homologous chromosomes and controls the synapsis and separation of homologous chromosomes until the first polar body is excreted (Sato and Tokmakov, 2020). This process consumes a large amount of ATP, so the distribution of mitochondria is of great importance during the meiosis process. More and more evidence suggested that stress-induced developmental competence impairment of oocyte involves alterations in mitochondrial functioning (Roth, 2018). From the perspective of cytobiology, we explored the effects of BPA treatment on the distribution of mitochondria in mouse oocytes. Our results showed that the rate of abnormal distribution of mitochondria was significantly higher in the group treated with 100 μM of BPA. The abnormally distributed mitochondria were no longer aggregated around the spindle; rather, they were clustered or dispersed in the oocyte. This may lead to an insufficient supply of ATP required for meiosis and thus result in the obstruction of oocyte development.

Endoplasmic reticulum is a membranous organelle in oocyte, which is associated with some of the functions of mitochondria (Friedman and Voeltz, 2011). The main function of the endoplasmic reticulum is processing cell proteins. Distribution of ER can affect the oocyte quality, for example, ER displayed a homogeneous distribution pattern throughout the entire ooplasm during development of oocytes and embryos from diabetic mice (C. H. Zhang et al., 2013). And similar, our results indicated that BPA exposure caused abnormal ER distribution in the cytoplasm. Certain endogenous/exogenous stimulation or changes in physical/chemical conditions may induce considerable expression of unfolded or misfolded proteins in the endoplasmic reticulum, which triggers endoplasmic reticulum stress and activates the unfolded protein response (Qi and Chen, 2019). GRP78 is one of the main molecular inducers of endoplasmic reticulum stress (Rana, 2020). A study by Asahi et al. (2010) found that BPA treatment induces endoplasmic reticulum stress in mouse hepatocytes and concurrently raises GRP78 expression levels. Based on these mitochondria-related studies, we further explored the endoplasmic reticulum function of mouse oocytes treated with BPA and measured the expression level of GRP78. We found that the expression levels of GRP78 in all oocytes treated with 100 μM of BPA increased significantly, and the majority of GRP78 molecules were distributed around the spindle. This suggests that the reproductive toxicity of BPA may induce endoplasmic reticulum stress in oocytes, which interferes with the normal function of the endoplasmic reticulum and disrupts the synthesis and processing of proteins needed for meiosis. This ultimately has an adverse impact on the maturation quality of oocytes.

The Golgi apparatus plays a central role in many intracellular transport events related to protein modification and delivery. After germinal vesicle breakdown (GVBD), the chromosomes are concentrated, and the Golgi apparatus is also fragmented; after that, in the MI oocytes, the Golgi apparatus was further broken and distributed around the spindle until the MII phase (Moreno et al., 2002). It has been reported that superovulation led to the decline of oocyte quality, one of the reasons is superovulation caused the distribution of Golgi apparatus changed significantly (Kalthur et al., 2016). We further explored the Golgi apparatus distribution in mouse oocytes treated with BPA, and the majority of Golgi apparatus distribution were around the spindle in MI period, but the GM130 protein expression was significantly decreased. GM130 protein participates in the maintenance of Golgi apparatus (Nakamura, 2010). Thus, we speculated that BPA exposure caused abnormal structure of Golgi apparatus but does not affect the distribution of Golgi apparatus in mouse oocytes.

Lysosomes play an important role in the intracellular vesicles transport system by digesting endogenous and exogenous macromolecules. They are membranous organelles that are closely related to the functions of the endoplasmic reticulum and mitochondria and are involved in autophagy (Miao et al., 2019). LAMP2 is a lysosomal marker protein. Its function is to protect the lysosomal membrane from digesting itself and maintain an acidic environment inside the lysosome. A study by Sun et al. (2020) showed that lysosomal damage can lead to an increase in LAMP2 levels. To explore the toxic effect of BPA on the intracellular organelles of mouse oocytes, we observed changes in LAMP2 expression levels in mouse oocytes as an index of lysosomal function. The results showed that the LAMP2 levels of oocytes treated with 100 μM of BPA increased significantly. A study by Han et al. (2016) suggested that increases in LAMP2 levels are related to autophagy. Taken together, our findings suggest that BPA treatment may lead to abnormal lysosomal function of oocytes, which triggers a lysosome-related autophagy reaction that interferes with the oocyte maturation.

## Conclusion

In conclusion, BPA treatment can disrupt the *in vitro* maturation of mouse oocytes. Specifically, BPA reduces the excretion rate of the first polar body. At the organelle level, this may be caused by the reproductive toxicity of BPA, which leads to the abnormal distribution of mitochondria, endoplasmic reticulum stress, abnormal Golgi apparatus structure, and lysosomal damage. These abnormal organelle functions lead to regional dysfunctions of the oocyte, which ultimately disrupts oocyte maturation.

## Data Availability Statement

The raw data supporting the conclusions of this article will be made available by the authors, without undue reservation.

## Ethics Statement

The animal study was reviewed and approved by the Institutional Animal Care and Use Committee of the College of Veterinary Medicine, Northwest A&F University (No. 2018011212).

## Author Contributions

M-HP and BM designed the study. M-HP and Y-KW performed the majority of the experiments. B-YL, HZ, CL, and J-LW contributed to the regents and materials. M-HP, BM, and L-LH analyzed the data. M-HP and BM wrote the manuscript. All authors contributed to the article and approved the submitted version.

## Conflict of Interest

The authors declare that the research was conducted in the absence of any commercial or financial relationships that could be construed as a potential conflict of interest.
